# End Treatment Response and Sustained Viral Response in Patients With Hepatitis C Virus Receiving Sofosbuvir and Daclatasvir

**DOI:** 10.7759/cureus.38833

**Published:** 2023-05-10

**Authors:** Attiya S Rahman, Muhammad Amir, Qaiser Jamal, Mehwish Riaz, Komal Fareed, Muhammad Siddiqui

**Affiliations:** 1 Department of Medicine, Abbasi Shaheed Hospital, Karachi, PAK; 2 Department of Medicine, Jinnah Postgraduate Medical Centre, Karachi, PAK; 3 Department of Medicine, Karachi Medical and Dental College, Karachi, PAK; 4 Department of Community Medicine, Foundation University, Islamabad, PAK; 5 Department of Research, Saskatchewan Health Authority, Regina, CAN

**Keywords:** sofosbuvir (sof), sustained viral response, end of treatment response, genotype 3, hepatitis c virus (hcv)

## Abstract

Objective

The main purpose of this study was to determine the end treatment response (ETR) and sustained viral response (SVR) in hepatitis C virus (HCV) patients receiving sofosbuvir and daclatasvir daily for 12 weeks.

Methods

This is a prospective open-label interventional study conducted from March 2018 to December 2020 in the outpatient departments of Abbasi Shaheed Hospital and Lyari General Hospital, Karachi. Patients with chronic infection of HCV, confirmed with ribonucleic acid (RNA) polymerase chain reaction (PCR) (qualitative analysis) were invited to participate in the study. All patients with positive HCV antibodies were evaluated clinically, with laboratory, and imaging assessment earlier to treatment. Statistical analysis was performed using SPSS version 20.0 (Armonk, NY: IBM Corp.).

Results

A total of 1043 patients participated in the study with a female predominance, 699 (67%) females. A majority (67.9%) of the study participants were aged between 15 and 45 years. After treatment of 12 weeks with sofosbuvir and daclatasvir 1039 (99.9%) patients achieved SVR while 1038 (99.6%) achieved an end treatment response. There was no significant association found between changes in alanine aminotransferase (ALT) levels, gender, and age among study participants.

Conclusion

Sofosbuvir and daclatasvir are found to be extremely effective for patients with hepatitis C in Pakistan. However, additional investigation including a larger sample size and involving a multicenter setting is recommended.

## Introduction

Viral infections (hepatitis A to E), alcohol intake, toxins, and metabolic dysfunction can result in hepatitis (inflammation of liver parenchyma) [1]. Infection with hepatitis C virus (HCV) has long been a major global public health issue; it is not only a prominent cause of end-stage liver disease, but it also increases death rates for a variety of extrahepatic disorders [2].

Approximately 58 million people have chronic HCV infection worldwide, with around 1.5 million new infections each year [3]. According to World Health Organization, around 290,000 individuals died from hepatitis C in 2019, primarily from cirrhosis and hepatocellular carcinoma (primary liver cancer) [3]. All regions of the World Health Organization (WHO) are affected by HCV. With 12 million chronically sick persons in each region, the Eastern Mediterranean and European regions have the largest illness burden [3]. With a 4-8% countrywide prevalence of hepatitis C, Pakistan has the second-highest burden in the world [4]. HCV elimination by 2030 was a commitment made by the World Health Assembly in 2016. To meet this challenging objective, 90% of all HCV patients must have a prompt diagnosis, and around 80% of all eligible patients must receive treatment with direct-acting antivirals [5].

Non-pegylated and pegylated interferons (PegIFN) therapy over the first two decades of chronic HCV therapy, as well as ribavirin, resulted in a decline in effectiveness and was ineffectively tolerated. During the period 2001-2011, depending upon the HCV genotype, PegIFN with ribavirin was the standard therapy for treatment. In genotype 1, the percentage of people who had a durable virological response ranged from 40% to 50% after 24 weeks or 48 weeks of combination treatment [6]. Even though pegIFN was more acceptable than non-pegylated forms, many patients were unable to tolerate PegIFN, and ribavirin frequently caused hemolytic anemia and other side effects. Issues concerning ribavirin's teratogenicity are also problematic, making treatment more difficult [7].

For patients older than 12 years of age, the WHO suggests therapy with pan-genotypic direct-acting antivirals (DAAs). Most people with HCV infection may be cured with DAAs, and treatment time is brief (about 12-24 weeks), depending on whether cirrhosis is present or not [3].

After endorsement by Food and Drug Administration in 2013, sofosbuvir a non-structural protein 5B (NS5B) inhibitor was the driving DAAs taken after daclatasvir which is an NS5A inhibitor [8]. By lowering IL-1 synthesis and NF phosphorylation, direct-acting antiviral medication treatment has been proven to diminish innate immune activation. This reduces inflammation, which in turn reduces fibrosis and damage in the liver. Direct-acting antiviral drug therapy can result in reduced expressions of interleukin CXCL10 and CXCL11 [1]. According to the ALLY 3+ study, the combination of sofosbuvir and daclatasvir in genotype 3 individuals is safe and effective, with an individual SVR of 92% in treatment-naive patients and 89% in the treatment-experienced patient [9]. The combination has few medication interactions and has been used safely in patients having liver transplants, kidney transplants, and HIV-co-infected individuals [10].

Combination therapy of interferon and ribavirin has been used in Pakistan for a long time. For this reason, much research is being done on this topic [11,12]. In recent years, direct-acting antivirals have been just introduced in Pakistan. As a result, there is little research that represents the effectiveness and responsiveness of the impacted community. Since this therapy is successful in almost all genotypes. The main purpose of this study was to determine the ETR and SVR in HCV patients receiving sofosbuvir and daclatasvir daily for 12 weeks.

## Materials and methods

**Figure 1:**
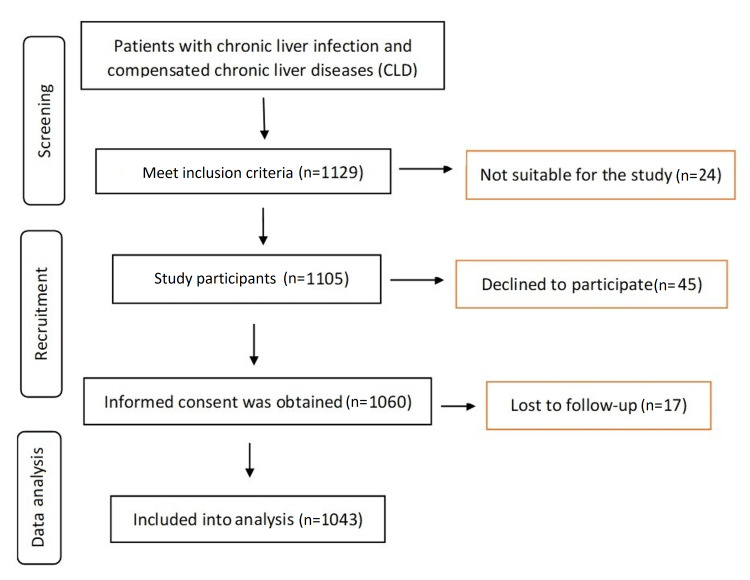
Flow diagram showing steps of the study.

The present study was an interventional study carried out in the Department of Medicine of Abbasi Shaheed Hospital and Liyari General Hospital between 2018 and 2020. A favorable ethical opinion was obtained (KMDC/2018) from Abbasi Shaheed Hospital and Karachi Medical and Dental College Research Ethics Board before the commencement of the study. Written informed consent was obtained from the study participants. All eligible participants had their identifiable data protected by assigning each participant a unique identifier (ID), which was only linked to their medical record number. The master list contained the study-assigned participant number and participant name only. De-identified data were entered during data collection. This database was stored with the principal investigator on encrypted and password-protected jumpdrives, to which only they had access. All other members of the research team were only able to view the study ID and the relevant information that is associated with it. A total of 1129 patients were contacted for prospective clinical research with chronic liver infection and compensated chronic liver diseases (CLD), which was confirmed with RNA polymerase chain reaction (PCR) (qualitative analysis) (Figure 1). Written consent was taken from all the participants. Out of the 1129 patients contacted for this study, 45 were unwilling to participate, 17 drop out at follow-up and 24 were excluded from the study because they did not meet the inclusion and exclusion criteria. Patients excluded from the study are those who have comorbid conditions, such as positive human immunodeficiency virus (HIV), positive hepatitis B surface antigen (HBsAg), other chronic liver diseases (CLD) namely, alcoholic liver disease, hepatotoxic drugs, autoimmune chronic hepatitis, and hemochromatosis and those with decompensated liver disease (such as variceal bleeding and ascites). All patients with positive HCV antibodies were assessed clinically, with laboratory, and imaging assessments before treatment.

Before therapy, all patients underwent a detailed medical history, including a history of past HCV treatment and any signs of decompensated cirrhosis, as well as a comprehensive medical examination. Baseline laboratory testing included a complete blood count, liver function tests, kidney function tests, HBsAg, a pregnancy test for females of childbearing age, and abdominal ultrasonography, as well as genotyping. The serum load of HCV was calculated quantitatively by performing real-time PCR, and it was repeated at the end of treatment at 12 weeks and after three months of treatment to detect sustained virological response.

Definitions and intervention

As per the American Association for the Study of Liver Diseases guidelines PCR-positive patients at the end of treatment and three months after the completion of treatment were stated as non-responders [13]. Patients who were negative by PCR at the end of treatment at 12 weeks but were found to be positive by PCR at three months after the end of treatment were labeled as relapsed. For 12 weeks before breakfast, treatment-eligible individuals received sofosbuvir 400 mg daily and daclatasvir 60 mg daily.

Study endpoint

The primary endpoint of our study was detecting the percentage of end-treatment response after 12 weeks and sustained virological response after three months. No adverse events were observed with the drugs. Hence, none of the patients had to stop the drug due to serious side effects.

Statistical analysis

Statistical analysis was accomplished using SPSS version 20.0 (Armonk, NY: IBM Corp.). Frequencies and percentages were calculated for sex, ETR, and SVR rate. Serum glutamic pyruvic transaminase (SGPT) was expressed as mean, standard deviation, and 95% confidence interval (CI). Chi-square tests and logistic regression analysis were used to assess the association between the ETR and SVR with sex, and age, and independent t-tests were used to compare the mean of SGPT between ETR and SVR groups.

## Results

A total of 1043 patients agreed to participate in the study. Overall, female predominance was noted in 699 (67%) females (Table 1). The female-to-male ratio was 2.03:1 (699/344). A majority (67.9%) of study participants were aged between 15 years and 45 years. The median baseline alanine aminotransferase (ALT) was 45 (interquartile range: 23).

**Table 1:** Frequencies and percentages of sex, age, SVR, and ETR. SVR: sustained viral response; ETR: end treatment response

Variables		Frequency	Percent
Gender distribution (n=1043)	Male	344	33.0
	Female	699	67.0
Age in years (n=1031)	12-14	17	1.6
	15-45	697	67.6
	45-65	317	30.7
SVR (n=1040)	Achieved	1039	99.9
	Not achieved	1	0.1
ETR (n=1042)	Achieved	1038	99.6
	Not achieved	4	0.4

No significant difference was observed (p=0.92; 95% CI: -97.04 to 87.63) between mean serum glutamic pyruvic transaminase in SVR who achieved (56.29±47.03) and not achieved (56.41±47.06). Similarly, No significant difference was observed (p=0.68; 95% CI: -42.69 to 64.42) between mean SGPT in SVR who achieved (56.41±47.06) and not achieved (45.33±2.89). Logistic regression and chi-square test results show no significant association between sex and SVR (p=0.669). Similarly, no significant association was observed between sex and ETR (p=0.598) (Table 2). Similarly, logistic regression and chi-square test results indicate no significant association between age and SVR (p=0.325). Likewise, no significant association was observed between age and ETR (p=0.931) (Table 3).

**Table 2:** Gender association with SVR and ETR. SVR: sustained viral response; ETR: end treatment response

Variables		Male	Female	Total	p-Value
SVR (n=1040)	Achieved	344 (100)	695 (99.9)	1039 (99.9)	0.669
	Not achieved	0	1 (0.1)	1 (0.1)	
ETR (n=1042)	Achieved	343 (99.7)	695	1038 (99.6)	0.598
	Not achieved	1 (0.3)	3 (0.4)	4 (0.4)	

**Table 3:** Age association with SVR and ETR. SVR: sustained viral response; ETR: end treatment response

Variables		Age in years			Total	p-Value
		12-14	15-45	45-65		
SVR (n=1028)	Achieved	17 (100)	694 (100)	316 (99.7)	1027 (99.9)	0.325
	Not achieved	0	0	1 (0.3)	1 (0.1)	
ETR (n=1030)	Achieved	17 (100)	693 (99.6)	316 (99.7)	1026 (99.6)	0.931
	Not achieved	0	3 (0.4)	1 (0.3)	4 (0.4)	

## Discussion

Pakistan has the world's second-highest HCV burden globally [14]. Despite the existence of generic direct-acting antivirals in Pakistan and the associated reduction in treatment costs, the incidence of hepatitis C remains stable, with no signs of diminution. One explanation for its continuation is the lack of a comprehensive, population-wide screening program capable of identifying the missing millions of persons in need of treatment [4]. This study showed very promising results, 1039 (99.9%) achieved sustained virological response while 1038 (99.6%) achieved ETR which is far superior to many other studies conducted worldwide. The combination therapy of sofosbuvir plus daclatasvir was well tolerated with few side effects.

A study conducted on 262 patients showed an ETR of 259 (98.9%) and sustained virological response in 248 (95.8%) patients when given a combination of sofosbuvir plus daclatasvir in genotype 3 patients [15]. Another study done by Butt on 125 patients depicted that ETR was achieved by 124 (99.2%) and SVR at 24 weeks was attained by 96 (95%) patients. Treatment-naive individuals and those without cirrhosis had better virological responses than treatment-experienced patients and those with cirrhosis [16].

A study conducted by Butt et al. showed ETR was achieved in 196 (98%) and SVR in 186 (93%) chronic hepatitis C (naïve) patients, respectively, who received sofosbuvir and daclatasvir [17]. Another study performed by Mushtaq et al. shows that in the duration of 12 weeks, 95.5% of patients attained SVR, whereas 96.8% of patients achieved ETR [18]. A randomized phase III study (ALLY-3+), a pivotal trial testing sofosbuvir and daclatasvir in genotype 3 patients, demonstrated an SVR12 of 90% (45/50) [9]. A study conducted by Tao et al. in a real-world cohort study showed ETR of 100% (57/57) and SVR of 85.96% (49/57) at 24 weeks after using the same combination of drugs [19]. A study conducted by Umar et al. in Iran also evaluated the results of generic sofosbuvir and daclatasvir in genotype 3 patients with SVR12 of 98% (40/41) [8].

Several factors, including bioequivalence of generics vs branded medications, compliance, study population, and probable underlying drug resistance, may be responsible for this disparity in outcomes [8]. Research from Iran also investigated the efficacy of generic sofosbuvir and daclatasvir in genotype 3 patients, and their results were significantly superior, with SVR12 of 98% (40/41) [8].

Kwo et al. also showed that sofosbuvir/daclatasvir therapy, with or without ribavirin, had high SVR12 rates (84%) and was usually well-tolerated in this patient population [20]. Furthermore, Ippolito et al. observed SVR12 rates of 97.6% in patients with advanced fibrosis (n=575) and 93.6-100% in patients with cirrhosis (n=2037), suggesting that DAAs may be administered in patients with advanced cirrhosis [21].

In this study, ALT remained the same for patients who attained ETR and SVR but an increase in ALT levels was observed who did not achieve ETR and SVR from 45.33±2.89 to 61.00±0 but the association was insignificant, but a study done by El Raziky et al. and El Kassas showed a decrease in ALT levels with significant association [22,23].

In this study, 344 (100%) male and 695 (99.9%) female patients achieved SVR, while 343 (99.7%) males and 695 (99.6%) females achieved ETR, however, there was no significant association was observed between gender and status of SVR and ETR. The results are similar to the study by El Raziky et al. [23] and Mushtaq et al. where no significant association was observed between the attainment of SVR concerning gender [18]. In this study increasing age does not depict any significant association between the achievement of SVR and ETR similar to the study done in the past but there was a significant association observed between decreasing age and attainment of SVR in a study done by Ahmed et al. [24] and Mushtaq et al. [18]. Another study done by El Kassas et al. showed age >50 years have a significant association with SVR [22].

In Pakistan, mainly from Punjab province, there have been various studies, reporting the efficacy of sofosbuvir-based therapies in HCV genotype 3-infected patients. It has been found that sofosbuvir-based dual or triple therapy has so far shown to be very effective in genotype 3 patients, with SVR after 24 weeks of treatment found to be 82.2-99.34% [25]. However, the results are suboptimal, especially in patients with a decompensated liver and with or without significant fibrosis [26-29]. So far, the largest study with a cohort of 1375 patients from Lahore performed from 2014 to 2016 has also shown a remarkable SVR rate of 97-99% in genotype 3-infected patients after sofosbuvir treatment as double or triple therapy regime which was similar to this study [25,30].

In this study, even with our effective study design, the study sample size was restricted to only the two tertiary care hospitals in the city of Karachi perhaps excluding a substantial population in other infirmaries and catchment areas. In the future, randomized control trials for longer follow-ups or a higher dose of treatment are needed before recommending it in our population due to its unseemly effects, and financial and observance-related implications.

## Conclusions

Daclatasvir and sofosbuvir is a highly efficient combination in individuals with chronic hepatitis C genotype 3 infection in Pakistan, with SVR12 of about 99.6% in genotype 3 patients. These non-proprietary medicines will be critical in eradicating hepatitis C infection in the underdeveloped globe. However, further investigation with a larger sample size, and longer follow-up involving a multicenter site is required.
